# Lateral thoracic artery aneurysm with lung abscess and empyema caused by *Streptococcus* *intermedius*

**DOI:** 10.1002/jgf2.448

**Published:** 2021-05-05

**Authors:** Tatsuya Fujihara, Naoya Itoh, Shuhei Yamamoto, Hanako Kurai

**Affiliations:** ^1^ Division of Emergency and Critical Care Department Shimane Prefectural Central Hospital Izumo Japan; ^2^ Division of Infectious Diseases Aichi Cancer Center Hospital Nagoya Nagoya Japan; ^3^ Division of Infectious Diseases Shizuoka Cancer Center Hospital Sunto‐gun Japan

**Keywords:** emergency medicine, hospital general medicine, infectious diseases

## Abstract

This is a case of pseudoaneurysm associated with lung abscess caused by *Streptococcus* *intermedius*. This infection can be fatal, as these bacteria can invade the vascular wall and induce lethal hemorrhage.

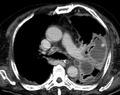

A 66‐year‐old man with hypertension presented to our hospital with acute chest pain, continuous cough, and fever for one month that began after an influenza B virus infection.

On examination, he appeared ill and was febrile (body temperature, 38.3°C). His vitals were as follows: blood pressure, 120/84 mmHg; pulse, 121 beats/min; respiratory rate, 25 breaths/min; O_2_ saturation, 94% on 6 L/min of O_2_; and Glasgow coma score, 15. Chest examination revealed decreased breath sounds on the left hemithorax. Cardiovascular and abdominal examinations were unremarkable.

Chest computed tomography (CT) showed a mass in the upper and middle zones of the left lung with pleural effusion. Thoracic drainage was performed, revealing that the effusion was purulent.

He was admitted after a diagnosis of a lung abscess complicated with pyothorax was made and was treated with intravenous ampicillin/sulbactam 3 g every 6 h. Despite drug treatment, he required endotracheal intubation and invasive mechanical ventilation due to the deterioration of his respiratory condition. On admission to the intensive care unit, additional intravenous vancomycin was administered until the pleural effusion culture results were obtained. *Streptococcus* *intermedius* was identified in the pleural effusion culture.

On the 4th day of admission, his respiratory and circulatory functions improved gradually; however, on the 7th day, he presented with a sudden onset of massive hemoptysis and thoracic bleeding. Just before the hemoptysis, a contrast‐enhanced CT (CECT) showed a middle lobe lung abscess with a strongly enhanced lateral area consistent with a pseudoaneurysm (Figure 1). We performed unilateral intubation of the nonbleeding lung and planned an immediate transcatheter artery embolism (TAE).

**FIGURE 1:**
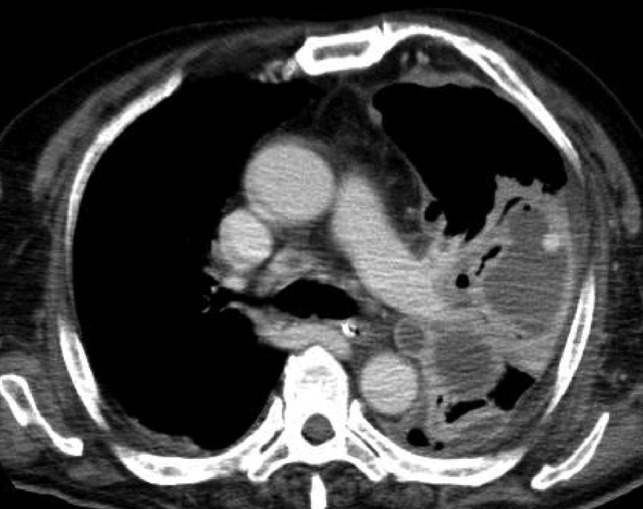
Contrast‐enhanced computed tomography shows a high‐density area within the lung abscess

Although he developed cardiopulmonary arrest due to hypoxemia and hypovolemia caused by massive hemoptysis during TAE, the procedure was performed after the return of spontaneous circulation (Figure 2). Unfortunately, the patient died on the 16th day of admission.

**FIGURE 2:**
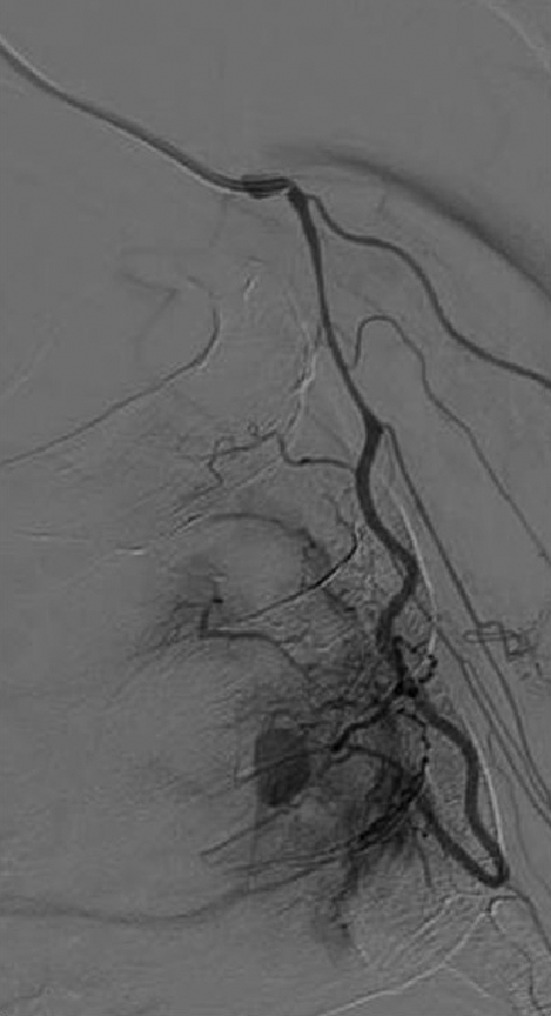
Angiography reveals a pseudoaneurysm in a branch of the lateral thoracic artery

The *S*. *anginosus* group is highly virulent, is rapidly progressive, and tends to cause abscesses.^1^, ^2^ Among the microbes in this group, *S*. *intermedius* is very prevalent in older adults and in patients with pneumonia or lung abscess with pleural effusion.^3^ In this case, a lung abscess caused by *S*. *intermedius* was complicated by a left thoracic artery pseudoaneurysm. The aneurysm ruptured and resulted in a fatal airway emergency due to hemoptysis and massive hemorrhage from the thoracic cavity.

Rasmussen aneurysm, which is a pseudoaneurysm that is associated with lung abscesses caused by tuberculosis, is well‐known; however, pseudoaneurysms caused by nontuberculous infections are rare.^4^ To the best of our knowledge, there is only one existing report of pseudoaneurysm‐associated lung abscess caused by this group of microorganisms.^4^ In addition, They can invade tissues, such as the major fissure of the lung, diaphragm, chest wall, and pulmonary arterial walls,^2^, ^4^ and may lead to lethal hemorrhage. Therefore, CECT at a lower threshold should be considered when lung abscesses caused by these bacteria are diagnosed or when symptoms, such as hemoptysis or bloody pleural effusion, are present.

## CONFLICT OF INTEREST

The authors have stated explicitly that there are no conflicts of interest in connection with this article.

## CONSENT

Written consent was obtained from the patient's family for the publication of this case report.
